# Multidisciplinary team meetings in treatment of spinal muscular atrophy adult patients: a real-life observatory for innovative treatments

**DOI:** 10.1186/s13023-023-03008-6

**Published:** 2024-01-24

**Authors:** Emmanuelle Salort-Campana, Guilhem Solé, Armelle Magot, Céline Tard, Jean-Baptiste Noury, Anthony Behin, Elisa De La Cruz, François Boyer, Claire Lefeuvre, Marion Masingue, Louise Debergé, Armelle Finet, Mélanie Brison, Marco Spinazzi, Antoine Pegat, Sabrina Sacconi, Edoardo Malfatti, Ariane Choumert, Rémi Bellance, Anne-Laure Bedat-Millet, Léonard Feasson, Carole Vuillerot, Agnès Jacquin-Piques, Maud Michaud, Yann Pereon, Tanya Stojkovic, Pascal Laforêt, Shahram Attarian, Pascal Cintas

**Affiliations:** 1 Service de Neurologie du Pr Attarian, Centre de Référence des Maladies Neuromusculaires PACA Réunion Rhône Alpes, Timone University Hospital, Aix-Marseille University, 264 Rue Saint-Pierre, 13385 Marseille Cedex 05, France; 2 Inserm UMR_S 910 Medical Genetics and Functional Genomics, Aix Marseille Université, Marseille, France; 3 Centre de Référence des Maladies Neuromusculaires AOC, Hôpital Pellegrin, CHU de Bordeaux, Bordeaux, France; 4 Laboratoire d’Explorations Fonctionnelles, Hôtel-Dieu, Centre de Référence des Maladies Neuromusculaires AOC, CHU de Nantes, Nantes, France; 5 Centre de Référence des Maladies Neuromusculaires Nord Est Ile de France, Lille, France; 6 Reference Centre for Neuromuscular Diseases AOC, University Hospital of Brest, Brest, France; 7 Centre de Référence des Maladies Neuromusculaires Nord/Est/Île-de-France, Institut de Myologie, Hôpital Pitié-Salpêtrière, AP-HP, Paris, France; 8 Centre de Référence des Maladies Neuromusculaires AOC, CHU et Université de Montpellier, Montpellier, France; 9 Pôle de Médecine Physique et de Réadaptation, Hôpital Universitaire Reims-Champagne-Ardenne, CHU Sébastopol, Centre de Référence des Maladies Neuromusculaires Nord Est Ile de France, Reims, France; 10 Nord-Est-Ile-de-France, Service de Neurologie, FHU Phenix, Centre de Référence de Pathologie Neuromusculaire, Raymond Poincaré University Hospital, Garches, APHP, Garches, France; 11 Centre de Réference des Maladies Neuromusculaires PACA Réunion Rhône Alpes Service de Neurologie, CHU de Saint-Etienne, Saint-Étienne, France; 12 Department of Neurology, Centre Hospitalier Universitaire d’Angers, Angers, France; 13 Service de Neurologie C, Hospices Civils de Lyon, Hôpital Neurologique Pierre Wertheimer, 69500 Bron, France; 14 Service d’Explorations Fonctionnelles Neurologiques, Hôpital Neurologique Pierre Wertheimer, 69500 Bron, France; 15 Service Système Nerveux Périphérique & Muscle, Centre de Référence des Maladies Neuromusculaires PACA Réunion Rhône Alpes, Centre Hospitalier Universitaire de Nice, Université Côte d’Azur, Nice, France; 16 APHP, Centre de Référence de Pathologie Neuromusculaire Nord-Est-Ile-de-France, Henri Mondor Hospital, University Paris-Est, Créteil, France; 17 Department of Rare Neurological Diseases, Centre de Référence des Maladies Neuromusculaires PACA Réunion Rhône Alpes, CHU de la Réunion, Saint-Pierre, France; 18 CeRCa, Site Constitutif de Centre de Référence Caribéen des Maladies Neuromusculaires Rares, CHU de Martinique, Hôpital P. Zobda-Quitman, Fort-de-France, France; 19 CHU de Rouen, Neurologie, Rouen, France; 20 Physiology and Exercise Laboratory EA4338, Centre de Référence des Maladies Neuromusculaires PACA Réunion Rhône Alpes, Rhône-Alpes Bellevue Hospital, University Hospital Center of Saint-Étienne, Saint-Étienne, France; 21 Service de Médecine Physique et Réadaptation Pédiatrique, Hôpital Femme Mère Enfant, Hospices Civils de Lyon, 69677 Bron Cedex, France; 22 Department of Clinical Neurophysiology, CHU Dijon Bourgogne, Dijon, France; 23 Department of Neurology, Nancy University Hospital, Nancy, France; 24 UVSQ, Paris-Saclay University, Paris, France; 25 Service de Neurologie, CHU de Toulouse Purpan, Place du Docteur Baylac TSA 40031, 8. Centre de Référence des Maladies Neuromusculaires AOC, 31059 Toulouse Cedex 9, France; 26 FILNEMUS, Marseille, France

**Keywords:** Spinal muscular atrophy, SMA, Multidisciplinary team meeting, Clinical decision-making, Treatment

## Abstract

**Background:**

In 2017, a new treatment by nusinersen, an antisense oligonucleotide delivered by repeated intrathecal injections, became available for patients with spinal muscular atrophy (SMA), whereas clinical trials had mainly involved children. Since 2020, the oral, selective SMN2-splicing modifier risdiplam has been available with restrictions evolving with time. In this peculiar context of lack of data regarding adult patients, many questions were raised to define the indications of treatment and the appropriate follow-up in this population. To homogenize access to treatment in France, a national multidisciplinary team meeting dedicated to adult SMA patients, named SMA multidisciplinary team meeting, (SMDTs) was created in 2018. Our objective was to analyze the value of SMDTs in the decision-making process in SMA adult patients and to provide guidelines about treatment.

**Methods:**

From October 2020 to September 2021, data extracted from the SMDT reports were collected. The primary outcome was the percentage of cases in which recommendations on validating treatment plans were given. The secondary outcomes were type of treatment requested, description of expectations regarding treatment and description of recommendations or follow-up and discontinuation. Data were analyzed using descriptive statistics. Comparisons between the type of treatment requested were performed using Mann–Whitney test or the Student t test for quantitative data and the Fisher’s exact test or the χ^2^ test for qualitative data.

**Results:**

Cases of 107 patients were discussed at the SMDTs with a mean age of 35.3 (16–62). Forty-seven were SMA type 2, and 57 SMA type 3. Twelve cases were presented twice. Out of 122 presentations to the SMDTs, most of requests related to the initiation of a treatment (nusinersen (n = 46), risdiplam (n = 54), treatment without mentioning preferred choice (n = 5)) or a switch of treatment (n = 12). Risdiplam requests concerned significantly older patients (*p* = 0.002), mostly SMA type 2 (*p* < 0.0001), with greater disease severity in terms of motor and respiratory function compared to requests for nusinersen. In the year prior to presentation to the SMDTs, most of the patients experienced worsening of motor weakness assessed by functional tests as MFM32 or other meaningful scales for the most severe patients. Only 12% of the patients discussed had a stable condition. Only 49/122 patients (40.1%) expressed clear expectations regarding treatment. The treatment requested was approved by the SMDTs in 72 patients (67.2%). The most common reasons to decline treatment were lack of objective data on the disease course prior discussion to the SMDTs or inappropriate patient’s expectations. Treatment requests were more likely to be validated by the SMDTs if sufficient pre-therapeutic functional assessment had been performed to assess the natural history (55% vs. 32%) and if the patient had worsening rather than stable motor function (*p* = 0.029). In patients with approved treatment, a-priori criteria to define a further ineffectiveness of treatment (usually after 14 months of treatment) were proposed for 67/72 patients.

**Conclusions:**

In the context of costly treatments with few controlled studies in adults with SMA, in whom assessment of efficacy can be complex, SMDTs are ‘real-world observatories’ of great interest to establish national recommendations about indications of treatment and follow-up.

**Supplementary Information:**

The online version contains supplementary material available at 10.1186/s13023-023-03008-6.

## Introduction

First introduced in the early 2000s in the field of oncology, multidisciplinary team meetings (MDTs) have proven their value in optimizing and standardizing the management and decision-making for cancer patients worldwide [1]. The practice of MDTs has gradually been extended to other specialties for which management is complex, particularly those involved in the field of rare diseases. The goal of the MDTs is to bring together health professionals involved in the management of a patient to optimize the diagnostic and therapeutic process. In France, under the guidance of successive Rare Disease Plans, MDTs have been extensively developed in the neuromuscular diseases reference centers network (FILNEMUS) (https://www.filnemus.fr/) to reduce diagnostic wandering and to homogenize the therapeutic management of these patients [2]. These objectives are particularly challenging in the absence of opposable guidelines, as in the case of oncology MDTs.

Spinal muscular atrophy (SMA) is an autosomal recessive motor neuron disease caused by loss of the survival motor neuron 1 gene (SMN1), resulting in severe and progressive muscular atrophy and weakness [3]. In 2017, the first treatment in SMA, the antisense oligonucleotide nusinersen [4], was approved by the FDA and EMA, without any age or motor disability restriction. In France, in the context of early access, nusinersen was granted a temporary authorization for use (ATU) in 2016 for the treatment of patients with SMA who have never reached an autonomous walking stage. In January 2018, the French Transparency Commission defined the conditions for access to the treatment for SMA types 1, 2 and 3: “nusinersen is indicated as first-line treatment for types 1 and 2, and is discussed on a case-by-case basis for types 3, taking into account walking ability”. This commission defined at the same time that “the indication of nusinersen and the decision to stop it should be taken in an MDT in the neuromuscular reference centers”. At that time, the efficacy of the treatment in adult patients had not been the subject of any prospective or retrospective studies. Then, many questions were raised to define the indications: What are the most appropriate indications for treatment in adults? Should only progressing patients be treated? For type 3 SMA patients, should only patients losing their ability to walk be considered for treatment? In addition, the MDT decisions should define, at the time of the initial decision, the criteria for treatment discontinuation. The lack of established discontinuation criteria in the literature and studies also raised many questions: when should the efficacy of treatment in adults be assessed? Is a patient with stable disease a responder? What assessment methods are the most appropriate for severe adult SMA?

**Fig. 1:**
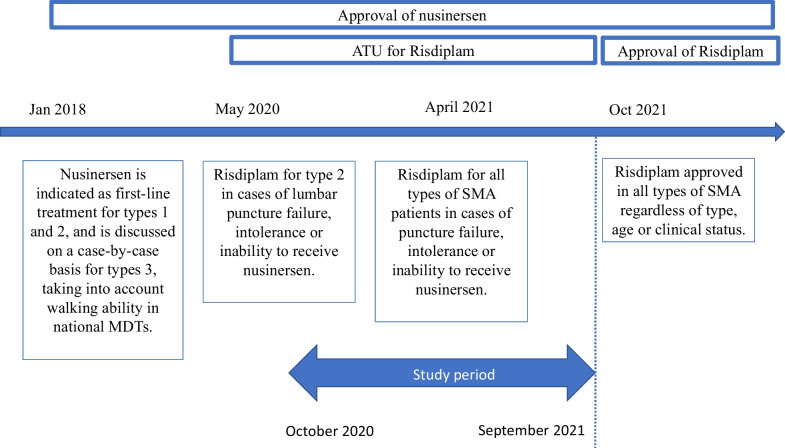
Changes in access to specific SMA treatments during the study period in France

In this context, the national neuromuscular network, Filnemus, proposed in 2018 the establishment of national MDTs dedicated to innovative therapies in adult SMA patients (SMDTs) inspired by those previously developed for the pediatric population in 2017 by the neuromuscular commission of the French Society of Pediatric Neurology [5]. The objectives were to homogenize the criteria for starting treatment, the follow-up and the evaluations of these patients. Afterward, risdiplam has been available under the ATU system since 2020, even though no comparative study between the two drugs was available. Access conditions to this treatment have changed over time (Fig. 1). Then in the period from 2020 to 2021, neurologists caring for adult SMA patients had to adapt to these different modalities of prescription and adjust their indications.

The aim of this study was to analyze the value of SMDTs in the decision-making process in SMA adult patients and to propose recommendations/guidelines about treatment (indications, choice, follow-up and criteria for discontinuation) derived from the SMDTs.

## Methods

### Description of the SMDTs

In France, patients (children and adults) affected by neuromuscular diseases are mainly managed by the neuromuscular reference centers of the Rare Disease network, Filnemus (https://www.filnemus.fr/la-filiere-de-sante-filnemus/presentation). Following a meeting of the Filnemus ‘Therapeutic Trials and Innovative Therapies Committee’ (https://www.filnemus.fr/la-filiere-de-sante-filnemus/les-commissions/essais-therapeutiques), a group of neurologists with expertise in SMA from each of the major French reference centers was set up. This group was formed to constitute a quorum of the SMDT. This quorum was defined to ensure the collegial nature of the SMDTs and to cover the entire French territory. Depending on the cases submitted to the SMDTs, experts from other medical disciplines were invited: pediatric neurologists, pulmonologists, physical medicine and rehabilitation physicians, radiologists. These experts from other disciplines also worked in a neuromuscular reference center or in close collaboration. An operational charter was defined and approved by all quorum members (Additional file 1). All doctors who occasionally present patient cases have signed and approved this charter. SMDTs could only be held if at least four neurologists of the quorum were attending. Formal SMDTs, including prospective record keeping, started in October 2018 for French adult SMA patients. The meetings were video-conferenced because of their frequency and national dimension using the dedicated tool Rofim (https://www.rofim.fr/). The meetings were initially held every 3 months and then monthly from October 2020. Patients must be informed prior to the SMDT by their referring physician. The referring physician agreed to obtain the patient's consent to the sharing and exchange of data. The discussions of the patient's case at the SMDTs were recorded in the patient's file.

To submit a case to the SMDT, a minimum set of clinical information was required to be provided by the referring practitioner (Additional file 2). In addition to medical data, nonmedical information such as psychosocial characteristics (occupation, family situation, social context, desire for pregnancy, etc.) was frequently required because of their impact on the decision-making process of a treatment plan. To assess the disease course and thus be able to define therapeutic objectives, an analysis of at least two functional assessments before submission to the SMDTs was needed.

### Therapeutic context in France during the study

Data for this study were collected from October 2020 to September 2021. The SMDTs have adjusted their decision-making process over time based on French health authorities. The French Health Authorities regulatory changes in access to SMA specific treatments during the study period are shown in Fig. 1.

### Decision making process

At the SMDTs, the patient's referring physician presents the patient's file to the quorum. Several information were requested:

- Reasons: introduction of a treatment, switch or any other issue
- Patient’s progression profile along with the elements of the functional assessment to support it
- Expectations/objectives regarding treatment impact

The discussion, supported by personal experience of the quorum and literature available at the time of the discussion, was to confirm the indication, define the appropriate means and period of evaluation, and define the criteria for discontinuation. A report was drawn up for each case during the SMDTs. If, at the end of the collegial discussion, no consensual solution was found, the report of the meeting specifies which clarifications are requested. For some patients, a second presentation to the SMDTs was requested.

### Data collection

The data collected retrospectively were age, age at onset, SMA type, number of copies of the SMN2 gene, walking ability, presence of a feeding tube, spine fusion surgery, noninvasive ventilation or invasive ventilation, reason for discussion of treatment to SMDTs, type of treatment requested if applicable, patients’ expectations if available, and type of functional tests performed.

In France, the functional evaluations performed routinely were the 32-item Motor Function Measure (MFM32) Motor Function Measure (MFM) [6], the Revised Upper Limb Module (RULM) [7], the 10-m walking test (10mWT) [8], the six-minute walking test (6MWT) [9], the Goal Attainment Scale (GAS) [10] and Myotools. Myotools are tools developed to assess upper limb strength and function in patients with neuromuscular diseases. Three of them assess pinch (MyoPinch), grip (MyoGrip), or wrist flexion and extension strength (MyoWrist) [11, 12]. Vital capacity (VC) was also collected. Patients with severe motor weakness of upper limbs were also evaluated by the clavitest (http://clavitest.free.fr/). This test provides a quantified assessment of keyboarding.

Several different types of major recommendations made during the SMDTs were collected: validation of starting a treatment, type of treatment, recommendations for follow-up assessment methods, frequency of patient assessment, a-priori criteria for discontinuing treatment, and need to resubmit the case to SMDTs.

### Design

The primary outcome of the study was the percentage of cases in which recommendations on validating treatment plans were given during SMDTs. The secondary outcomes were as follows: type of treatment request, description of expectations regarding treatment and description of recommendations for follow-up and discontinuation.

### Statistical analysis

Quantitative data were expressed as mean or median with ranges and compared using the Mann–Whitney test or the Student t test. Qualitative data were expressed as number and percentage and were compared using the Fisher's exact test or the Chi2 test. Statistical analysis were performed using IBM SPSS statistics (version 20). A two-tailed *p* value < 0.05 was considered significant.

### Ethical

This study was classified as a service evaluation, and therefore, no formal research ethics committee approval was needed. However, the study was conducted according to research governance guidance and in compliance with the Declaration of Helsinki.; that is, participants in the focus groups were given written and verbal information and time to ask questions prior to participation. They were assured of anonymity; participation was implicit of consent. All data from this study are anonymized and stripped of all sensitive personal and patient identifiers.

## Results

### Recommendations given by SMDTs

During the period from October 2020 to September 2021, 107 patients were discussed at the SMDTs (50 women (47%); mean age = 35.3 year (16–62)). Patient characteristics are detailed in Table 1. Forty-seven (44%) were SMA type 2, and fifty-seven were SMA type 3. The remaining three patients were type 1 (n = 1) and type 4 (n = 2). Most patients were presented only once, and 12 patients were presented twice; thus, 122 cases were discussed. Most of them were discussed for a decision to start treatment (n = 117). In 46 cases, the treatment requested was nusinersen; in 54 cases, risdiplam; and in 5 cases, request to initiate treatment without mentioning preferred choice. In the remaining 12 cases, a switch of treatment (nusinersen to risdiplam or risdiplam to nusinersen) was requested.

**Table 1:** Patients’ characteristics

	SMA type 1 (n = 1)	SMA type 2 (n = 47)	SMA type 3 (n = 57)	SMA type 4 (n = 2)
Mean age in years (range)	44	33.3 (20–55)	37.4 (16–62)	48 (40–56)
Mean age at onset (y)	NA	NA	7.2	29.5
Mean SMN2 copy numbers	MD	2.9	3.3	4
Wheel chair bound (%)	1 (100)	47 (100)	31 (54)	0 (0)
Feeding tube	1	3	1	0
Spine fusion surgery (%)	1 (100)	41 (87.2)	18 (31.6)	0
Noninvasive ventilation/invasive ventilation	0/1	22/10	7/2	0/0
Median MFM32 (% total score)	NA	12 (0–40.6)	56.0 (14.6–83.0)	83 (MD)
Median 6MWT (meters) (range)	NA	NA	215 (0–450)	400
Median 10mWT (s) (range)	NA	NA	9.0 (0–36)	MD
Median RULM (range)	NA	7 (0–18)	17 (2–37)	MD
Median FVC (%) (range)	NA	25 (1.1–54)	93 (46–140)	MD

*MD* missing data; *MFM32* 32-item motor function measure; *NA* not applicable; *RULM* revised upper limb module; *SMA* spinal muscular atrophy; *6MWT* six-minute walking test; *10mWT* ten-meter walking test

### Type of treatment requested

**Fig. 2:**
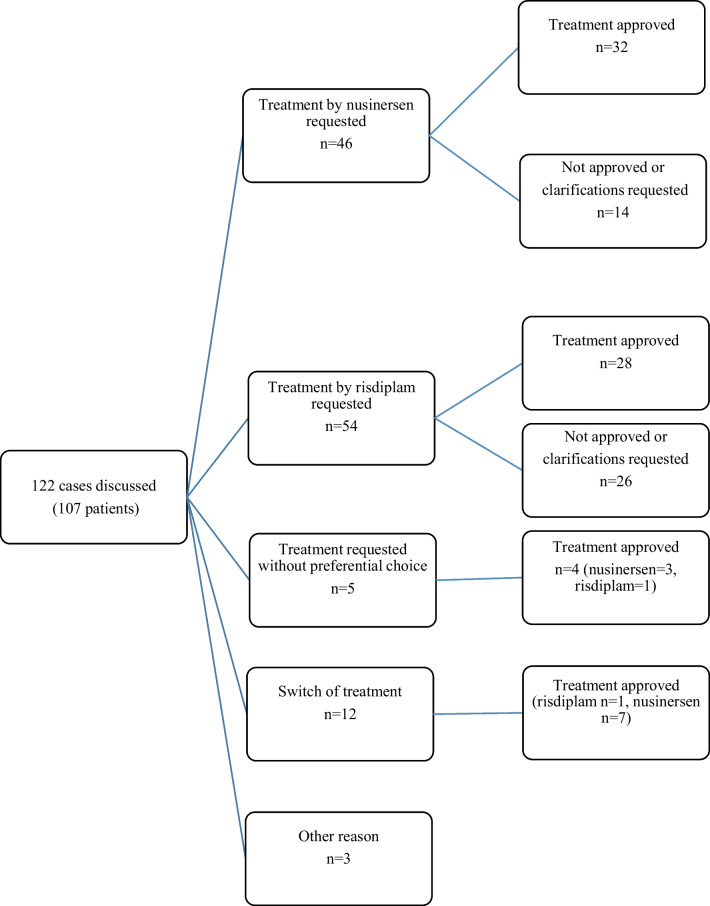
Distribution of cases presented at the SMDTs according to the reason for discussion

Figure 2 is an illustration of the treatment requests presented at the SMDTs. Nusinersen was requested in 46 cases (38%), mostly for SMA type 3 patients (n = 40, 87%). The remaining patients were type 2 (n = 5) and type 4 (n = 1). Treatment by Risdiplam was requested in 54 cases (44%), mostly for type 2 patients (n = 37, 69%). The remaining patients were type 3 (n = 16) and type 1 (n = 1). In 5 patients, treatment initiation was requested without mentioning the preferential drug by the referring physician. In 12 cases, a switch of treatment was requested. For 11 of them, it was a switch from nusinersen to risdiplam. The duration of prior treatment with nusinersen was highly variable (mean = 17.5 months; 0.5 to 42 months). Reasons for switching from nusinersen to risdiplam were difficulties related to the intrathecal injection procedure (pain (n = 3); access failure (n = 1); postlumbar puncture syndrome (n = 1)), organizational difficulties in performing intrathecal injections in the context of the COVID-19 pandemic (n = 1), and treatment ineffectiveness (n = 5). In one type 2 patient, a switch to nusinersen was requested after an 18-month treatment with risdiplam. The motivation for the switch was to obtain an improvement in motor function, whereas the patient was stable under nusinersen treatment.

When comparing subgroups of patients according to the type of treatment requested, risdiplam requests concerned significantly older patients (*p* = 0.002), mostly SMA type 2 (*p* < 0.0001), with greater disease severity in terms of motor and respiratory function compared to requests for nusinersen. Table 2 shows the main differences between the two groups.

**Table 2:** Comparison between subgroups of patients by type of treatment requested (nusinersen versus risdiplam)

	Request for nusinersen (n = 46)	Request for risdiplam (n = 54)	*P* value
Age at inclusion	**39.45**	**32.63**	**0.002**
Men	25	22	0.272
MFM32 (n)	**65.54 (n = 13)**	**24.80 (n = 10)**	**0.002**
Type 2: n (%)	**3 (7.1%)**	**30 (65.2%)**	**0.000001**
Type 3: n (%)	**37 (88.1%)**	**15 (32.6%)**	**0.00001**
Walking with aid (%)	**12 (28.6% = **	**3 (6.5%)**	**0.06**
Wheel-chair bound (%)	**23 (54.8%)**	**39 (84.8%)**	**0.012**
Eating alone (%)	**29 (85.3%)**	**15 (38.5%)**	**0.00001**
Eating with help (%)	**4 (11.8%)**	**12 (30.8%)**	**0.00001**
Depending on food (%)	**0 (0.0%)**	**8 (20.5%)**	**0.0001**
Feeding tube (%)	**1 (2.9%)**	**4 (1.3%)**	**0.00001**
Spine fusion surgery (%)	**10 (25.0%)**	**35 (81.4%)**	**0.0001**
No ventilation (%)	**35 (85.4%)**	**16 (36.4%)**	**0.0001**
Non invasive ventilation (%)	**6 (14.6%)**	**18 (40.9%)**	**0.0001**
Invasive ventilation (%)	**0 (0.0%)**	**10 (22.7%)**	**0.0001**

Significant differences between the two groups are indicated in bold (*p* < 0.005)

### Indications regarding treatment

**Fig. 3:**
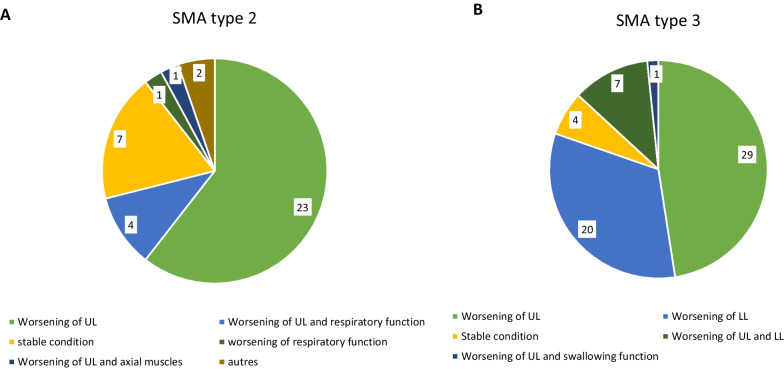
Reason for treatment request regarding SMA type. The diagram illustrates the patients' motor progression in the period prior to presentation to the SMDTs.** A** in SMA type 2 patients.** B** in SMA type 2 patients. UL: upper limbs, LL: lower limbs

Figure 3 summarizes the indications for considering treatment according to SMA subtypes. Most patients were being referred for upper or lower limb worsening of motor weakness. Only 12% of patients discussed at the SMDTs had a stable condition.

### Assessment of patients before treatment

Functional assessment was required before the case was presented to the SMDTs. The most frequently performed outcome measures were MFM (66.4%), FVC (29.5%), RULM (22.9%), 6MWT (14.7%), Myotools (13.1%), 10MWT (9.8%), TUG (2.4%), and Clavitest (4.1%). Forty-nine patients (45.7%) had the same functional tests performed twice with at least 6 months of follow-up before SMDTs (median follow-up prior to SMDTs of 11.5 months).

### Patient expectations regarding treatment

**Fig. 4:**
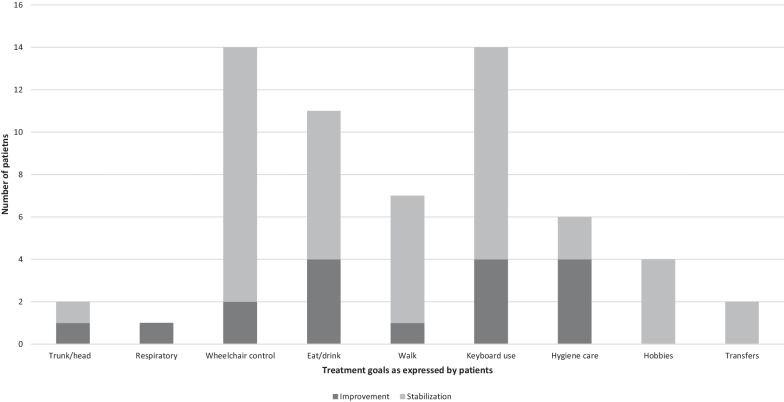
Patients’ expectations. This figure shows patients’ expectations of treatment in terms of function. The bars show the absolute number of patients who expressed an expectation for that function. Dark grey indicates expectation of improvement, light grey indicates expectation of stability

The description of the patient’s expectations is shown in Fig. 4. Only 49 patients (45.8%) expressed clear expectations regarding treatment before presentation to the SMDTs. Thirteen patients (26.5%) were waiting for an improvement mainly regarding their ability to eat or perform their hygiene care at least partially. Of the 34 requests for stabilization (69.4%), these mainly concerned their ability to control the wheelchair, use a keyboard for environmental control, recreational activities or their work. Two patients (4.0%) wished to stabilize their upper limb function and improve their trunk posture, especially when using a wheelchair.

### Analysis of SMDTs recommendation

The SMDTs approved treatment in 72 cases (67.3%) during the study period, with the following distribution: 69.5% for nusinersen requests, 51.8% for risdiplam, 80% for treatment without precision and 58.3% for a switch of treatment (Fig. 2). For nusinersen, treatment was not approved or clarification was requested by the SMDT quorum in 14 cases due to missing data on baseline functional assessment (n = 6, 13%) or patient expectations (n = 3, 7%), lack of evidence of disease progression (n = 7, 15%), or intercurrent disease (somatoform disorder, metastatic colorectal cancer) (n = 2, 4%). For risdiplam, treatment was not approved or clarification was requested by the SMDT quorum in 26 cases: no contraindications to intrathecal administration, very severe muscle weakness with difficulty measuring changes in functional assessment, and intercurrent disease (chondrosarcoma, unexplained inflammatory syndrome). Treatment by nusinersen was validated in 3 patients and treatment by risdiplam in one for request of treatment without precision. For the switch request, the change of treatment was not validated by the quorum due to a short-term pregnancy project (n = 1) and lack of data on progression under the first treatment used (n = 3).

Treatment requests were more likely to be validated by the SMDTs if sufficient pre-therapeutic functional assessment had been performed to assess the natural history (55% vs. 32%; *p* = 0.05) and if the patient had worsening rather than stable motor function (*p* = 0.029).

### Recommendations for patient follow-up

According to the initial objectives of the treatment and the patient's disease progression, different monitoring schemes were defined: at 6 and 14 months (66%), at 4, 8 and 14 months (12.3%), at 3 and 6 months (7.4%), and at 12 months (11%). The tests proposed for the follow-up included MFM (85%), RULM (10%), Myotools (39%), Clavitest (17%), 6MWT (21%), 10mWT (20%), and GAS (11%).

In patients on approved treatment, a priori criteria for discontinuation were defined in 67/72 patients. For the majority of patients, it was recommended that treatment efficacy be assessed at 14 months and that the patient be readmitted to the SMDT if treatment failure was suspected. In all but one case, the criteria for stopping treatment were worsening of a functional test and/or loss of a specific function (walking, dressing).

## Discussion

This study analyses the implementation of a collegial decision-making process in a context of innovative therapeutics for which little or no data were initially available. It offers a decision-support model when two innovative therapies are available without sufficient hindsight to assess their comparative benefits. This study demonstrates the evolution of therapeutic indications as collective feedback and real-life data become available. This decision-making methodology highlighted the need to be as precise as possible about patients' expectations, to adapt indications and, above all, evaluation methods to these expectations. It also demonstrated the need for a prior evolutionary slope, enabling us to define the criteria for discontinuing treatment as soon as the treatment decision is taken. Last but not least, this approach has made it possible to standardize patient decision-making and assessment procedures at national level, depending on their stage of disability and treatment decision.

The time period of our study reflects a particularly challenging point in the decision-making process for the treatment of SMA patients. At that time, nusinersen was approved for all types of SMA, while risdiplam was just becoming available under the ATU, with access conditions evolving over the period. From October 2020 to September 2021, treatment initiation requests accounted for 87.5% of the reasons for presenting to the SMDTs, including 38.3% of requests to start nusinersen, 45.0% of requests for risdiplam and 4.1% of requests for treatment without precision, the type of which was left to the discretion of the SMDT quorum. Ten percent of requests were for a switch of treatment. The higher proportion of requests for risdiplam was probably due to the recent availability of risdiplam at the time of the study and the convenience of its route of administration (oral vs intrathecal).The majority of requests for nusinersen were from type 3 patients, whereas the majority of requests for risdiplam were from type 2 patients. This difference is easily explained by the regulatory context of the period, as only patients with a contraindication to nusinersen were eligible for risdiplam.

Treatment was approved by the SDMTs in 72 patients (67.3%), with a higher proportion of nusinersen approvals than risdiplam approvals (69.5% vs. 51.8%). The reasons why the quorum of SDMTs issued a negative opinion or a need for clarification on a treatment request varied by treatment type. The majority of refusals for risdiplam were motivated by the absence of contraindications to intrathecal administration, which at the time of our study was a necessary criterion for obtaining an ATU, whereas refusals for nusinersen were mainly motivated by the inability to assess the future efficacy of the drug due to the lack of an evolutionary slope prior to presentation to the SMDTs or the overall stability of the disease.

Analysis of the percentage of cases with treatment validation reflects the decision-making process of the SMDTs. The rate of validated cases was higher when the pre-treatment evolutionary slope was defined, when motor deterioration was highlighted and when patient expectations were well defined. The assessment of the pre-treatment evolutionary trajectory was an important point in the SMDTs' discussions. In order to decide whether a treatment is ineffective or not, it is essential to have previously defined the criteria that define its effectiveness. In adult SMA, there is great heterogeneity in the profiles of disease progression [13, 14]. The definition of a responder patient thus requires prior availability of slopes on the main scales (MFM32, HFMSE, RULM). The example of late-onset Pompe disease, another genetic neuromuscular disease, has shown that in the absence of an initial definition of the individual evolutionary profile, recognizing the therapeutic benefit of the treatment is challenging in the presence of stability of the disease or of a slight worsening despite treatment [15]. The long-term efficacy of nusinersen into adulthood remains to be demonstrated. Only real-life data are available on nusinersen in adult type 3 SMA patients, and no published data on treated patients with type 4 SMA are available. Available retrospective and prospective observational studies as well as meta-analysis in adults indicate a mild treatment effect or a stabilization of motor function [16–21]. Concerning risdiplam, no clinical trials in adult patients have been conducted, although published clinical trials of risdiplam have included some adult patients [22]. In a monocentric longitudinal cohort study, HFMSE and Children's Hospital of Philadelphia Infant Test of Neuromuscular Disorders (CHOP INTEND) scores increased in parallel with increases of CMAP amplitudes in both median nerves in 18 adult patients with SMA type 2 or 3 treated with risdiplam during a 10-month treatment period [23]. Observational studies, meta-analysis and real life will likely define its therapeutic effect in adult patients [24–26].

Several functional scales have been validated in adult SMA [27–29]. In most cases, they enable the progression profile of the disease to be determined. However, in the most severe patients, these tests are affected by a floor effect, making it impossible to accurately assess the functional changes perceived by patients. In order to present a patient to SDMTs, it was mandatory to assess motor function using functional scales prior the discussion. The most commonly used were MFM32, RULM, 6MWT and myotools. The MFM32 is a functional scale widely used in spinal muscular atrophy. In the phase 3, double-blind, randomised, placebo-controlled trial of risdiplam, the primary endpoint was the change from baseline in the total score of the 32-item Motor Function Measure at month 12 [22]. This scale is more widely used in France than the HMFSE. For this reason, the MFM32 was preferred for routine patient follow-up. However, these scales have been shown to be equivalent [30]. However, only 45.7% had the same functional tests performed twice with a minimum follow-up of 6 months prior SMDTs. These values reflect the difficulties in assessing the most severe patients, as well as the presence of many patients who were lost to multidisciplinary consultations before the era of therapies. Once treatment initiation had been validated, the SMDTs provided recommendations on follow-up modalities. Over the course of the meetings, a consensus on functional assessment was gradually reached. For less severe patients, the “classic” assessment scales were proposed (MFM32, RULM, 6MWT). In the most severe forms, where a precise assessment was not possible because of the floor effect of the scales, personalized assessments based on the patient's main expectations were proposed: timed computer typing tests such as clavitest, wheelchair driving tests… In all cases, an assessment of the patient's reported outcome was proposed, focusing on expectations, by means of MCRO and/or GAS (Goal Attainment Scaling), especially in the context of patients with severe disease [31].

The analysis of patients' expectations of the treatment is an essential element in ensuring patient adherence and satisfaction. Adult SMA patients have a long experience of disability and progressive disease. Then, patients expect that a new treatment that could stabilize their disease represents more than 80% important progress [32, 33]. In our study, 45.8% of patients expressed clear expectations regarding treatment before presentation to the SMDTs. A very interesting point is that patients were waiting more for stabilization than improving their function (69.4% vs 26.5%). Patient expectations are focused on maintaining functions representing a major impact on their quality of life. Current outcome measures, which have been used in clinical trials, do not allow the assessment of patient expectations, and the experience of SMDTs outlined the importance of introducing patient-reported outcomes for the assessment of the benefits of treatments such as MCRO or GAS [34]. Thus, stabilization or improvement in their abilities for “use restroom without assistance”, “bed to chair transfer without assistance”, and “use a keyboard” represent the most important outcomes of a treatment and the reason for its continuation [35]. A great number of medico-social parameters contribute to the precise scope of patients' expectations. A careful definition of the patient's expectations at the time of indication is a prerequisite for establishing therapeutic goals and, consequently, discontinuation criteria.

To our knowledge, criteria for the discontinuation of treatment in adult SMA patients have not been defined in previous studies. However, while two expensive treatments are available in adults and their long-term safety and efficacy are not known, it seems essential to define criteria for discontinuing treatment and to carefully consider the indications. For this purpose, the SMDTs defined criteria for the discontinuation of treatment for the vast majority of patients at the time of validation of treatment. These criteria were defined on an ad hoc basis, based on the advice of quorum experts, taking into account the functional assessment performed prior to the SDMTs, the disease progression (worsening or stability) and most importantly the patient’s expectations. For most of the patients, the criteria for discontinuation of treatment were evidence of a deterioration on a functional test or the loss of motor function. Since the end of our study period, SMDTs have refined the criteria for cessation of treatment according to the minimal clinically important difference (MCID) that represents meaningful changes for patients. The used MCIDs for HFMSE, MFM32, RULM and 6MWT were 3 points, 3 points, 2 points and 30 m, respectively [27, 36]. The SMDTs used these MCIDs for a one-year period. However, in the future, it will probably be necessary to use criteria more suitable to adult SMA patients, integrating the specific features of different subtypes of SMA patients [37].

With increasing treatment options, the question of switching therapies is raised relatively frequently, even though no guidelines are available on this subject. Among the switch requests presented to the SMDTs, the majority (11/12) consisted of switching from nusinersen to risdiplam. This may be attributed to the fact that risdiplam had become available in adults at the time of our study. The switch motivation was mainly related to difficulty with the mode of administration of nusinersen. In the remaining 5 cases, the reason for the switch request was treatment failure. To date, there are very few data concerning switching therapies in SMA patients. These are mainly small studies of SMA type 1 or 2 children who received nusinersen first followed by gene therapy or of patients who received gene therapy and were then treated with nusinersen or risdiplam [38–40]. In an observational study of compassionate use of risdiplam in both type 1 and 1 adult and children patients with spinal muscular atrophy in Germany, some patients had previously received nusinersen. A total of 30.6% of type 1 and 12.2% of type 2 patients received risdiplam for loss of efficacy of nusinersen. However, the definition of loss of efficacy was not clearly defined [41].

Several limitations of our study are worth discussing. First, our study is a retrospective study based on the written reports of the SMDTs. SMDT meetings were not tape-recorded, which may have limited the comprehensiveness of the data collected. Second, the analysis of the responses of the SMDTs showed some variability that may be explained by different intrinsic and extrinsic factors, such as the different composition of the participants attending the SMDTs, the weight of the cumulative experience, and changes over time in regulatory aspects of access to treatment. Other studies exploring the decision-making process in MDTs have shown that patients’ nonmedical characteristics may influence challenging medical decisions [42]. However, because of the retrospective design of our study, it was not possible to assess the impact of this factor on the decision process. Finally, it would be interesting to have the patient's point of view and an analysis of the social benefits of the impact of these decisions, but the design of our study does not allow this.

## Conclusion

In SMA adult patients, there are still major issues for both available treatments, i.e., nusinersen and risdiplam, concerning adverse effects, the lack of evidence of their efficacy in the more severe forms of the disease and data on long-term effects. There is also no consensus about when and how to assess disease progression under treatment, as most of the clinical trials have focused on the pediatric population. Functional scales used in treated children are sometimes unsuitable for very severe adult patients. These issues have led the French neuromuscular network to consider personalized treatment according to the characteristics of each patient. The SMDTs constitutes a real-life observatory of the implementation of innovative treatments in SMA. The discussions during the SMDTS were of great interest to establish national recommendations about indications of treatment and follow-up. The analysis of the SMDTS revealed a heterogeneity of recommendations over time. This may be explained by the fact that MDT is a dynamic organization (physicians present, regulatory aspects regarding access to treatments, accumulation of experience). An analysis of the decision-making process is needed to improve the quality and homogeneity of decisions.

### Supplementary Information

**Additional file 1**. Operating charter of the national multidisciplinary team meetings dedicated to innovative therapies in adult SMA patients.

**Additional file 2**. Minimum set of clinical information form required to submit a case to the SMDT.

## Data Availability

All aggregated presented data in the manuscript were extracted from SMDTs report form (example provided in supplementary data). All SMDTs form are available in Rofim (www.rofim.fr/).Rofim is a data platform for authorized hosting of personal health data according to European General Data Protection Regulation. Raw data are not publicly available to preserve individuals’ privacy under the European General Data Protection Regulation.

## Abbreviations

ATU: Authorization for temporary use
GAS: Goal attainment scale
MFM: Motor function measure
MDT: Multidisciplinary team meeting
SMA: Spinal muscular atrophy
SMDT: Multidisciplinary team meeting dedicated to adult SMA patients
RULM: Revised upper limb module
6MWT: Six-minute walking test
10mWT: Ten-meters walking test
VC: Vital capacity
