# Supplementary material for: Multidisciplinary team meetings in treatment of spinal muscular atrophy adult patients: a real-life observatory for innovative treatments
Source: Orphanet J Rare Dis. 2024 Jan 24;19:24. doi: 10.1186/s13023-023-03008-6 (PMC10809505; doi:10.1186/s13023-023-03008-6)
Supplement: Supplementary file 1 — Additional file 1. Operating charter of the national multidisciplinary team meetings dedicated to innovative therapies in adult SMA patients. [file 13023_2023_3008_MOESM1_ESM.pdf]

filière de santé maladies rares / Filnemus Filière Neuromusculaire	RCP Thérapies innovantes pour les patients adultes porteurs d'une amyotrophie spinale	Version du 06/04/2021
**Charte de fonctionnement des Réunions de Concertation Pluridisciplinaire (RCP)**		
Groupe de travail :<br>Dr Emmanuelle CAMPANA-SALORT, Mme Mélanie BRISON		

I. Préambule

La présente charte a pour objectif de décrire l'organisation et le fonctionnement des Réunions de Concertation Pluridisciplinaire (RCP) de la filière de santé FILNEMUS (Filière de Santé Maladies Neuromusculaires). Les RCP sont destinées à tous les médecins qui suivent des patients atteints d'une pathologie neuromusculaire rare en lien avec la Filière FILNEMUS, sur tout le territoire national (métropole et outre-mer). Elles permettent aux patients de bénéficier de propositions diagnostiques et thérapeutiques basées sur l'avis d'experts du domaine, fondées sur leur expérience, les données les plus récentes de la littérature médicale et les connaissances de la science fondamentale.

II. Objectif de la RCP

L'objectif de cette RCP est de définir une proposition de prise en charge individualisée, la mieux adaptée à chaque patient, pour la mise en route, le suivi ou l'arrêt de thérapies innovantes dans le domaine des maladies neuromusculaires selon des critères de qualité évaluables. Ces propositions de prise en charge s'appuient sur les recommandations nationales (PNDS lorsqu'ils existent) et en leur absence, sur les recommandations des Sociétés Savantes et les référentiels nationaux.

Afin de permettre à tous les patients atteints de maladies neuromusculaires rares de bénéficier de la meilleure prise en charge possible, ces RCP sont proposées en web-conférences via l'outil ROFIM. Cet outil est accessible en se connectant au lien suivant : https://rofim.doctor/. L'accès aux séances de RCP se fait en acceptant l'invitation lancée par l'administrateur ou en demandant à participer à la RCP directement sur l'outil. La demande sera ensuite acceptée par l'administrateur. Cet outil web est accessible depuis n'importe quel poste équipé d'une connexion internet mais également depuis un téléphone portable en téléchargeant l'application. L'accès aux utilisateurs est protégé avec un code reçu par SMS ou email.

III. Principes de fonctionnement

Le Quorum

Le *quorum* permet d'assurer le caractère collégial de la RCP, il s'agit d'un critère-qualité essentiel à la tenue de la RCP. Le *quorum* est composé d'un nombre minimal de 4 experts définis selon une liste

définie par les coordonnateurs de la RCP. Des membres de spécialités différentes seront sollicités, en fonction du thème de la RCP (thème définit par les cas cliniques proposés).

Le quorum doit être respecté pour que l'avis formulé en RCP soit valide. En l'absence de quorum, la RCP sera déclarée invalide. Les membres du quorum qui se sont engagés pour une date doivent être présents à la séance ou se faire remplacer par un autre membre du quorum.

Les membres du quorum sont désignés selon leur expertise dans le domaine de la thérapie innovante impliquée et leur participation régulière à la RCP.

Coordination de la RCP

Le médecin coordonnateur (dénommé sur l'outil ROFIM médecin référent) est garant de la bonne organisation et du bon déroulement de la RCP. Il est présent pendant la réunion et résume l'avis collégial de chaque dossier à la fin de la discussion.

Professionnels participant aux RCP

La RCP est ouverte aux médecins spécialistes issus des CCMR et CRMR membres de la filière FILNEMUS.

Le médecin référent du patient participe à la RCP, ou est remplacé en cas d'impossibilité par un des médecins de l'équipe prenant en charge le patient.

Toute discussion en RCP s'effectue dans le cadre du secret professionnel. Chaque professionnel de santé s'engage à respecter cette règle de confidentialité. Les personnels non-médicaux ou non-paramédicaux qui souhaitent participer ces RCP devront nous en informer avant la séance et devront nous retourner un engagement de confidentialité signé. L'identité des participants est tracée sur la fiche RCP.

Des auditeurs libres silencieux (internes, étudiants en médecine, professionnels médicaux ou paramédicaux, chercheurs, etc..) peuvent participer à ces séances de RCP et sont également soumis à une clause de confidentialité.

Fréquence

La RCP a lieu une fois par mois. Elle se tient en web-conférence de 17h30 à 19h30 (heure métropolitaine), le premier jeudi de chaque mois généralement.

Toutes les dates de RCP sont planifiées à l'avance et ré-annoncées en amont de la réunion afin d'établir le quorum et recueillir les dossiers patients qui seront vus en RCP. Les calendriers des RCP sont disponibles sur le site internet de la filière FILNEMUS.

Dossiers concernés

- Patients adultes atteints d'amyotrophie spinale de type 1, 2 ou 3 confirmés sur le plan moléculaire
- Patients traités ou non
- Discussion de mise au traitement, d'effets indésirables, de suivi ou d'arrêt de traitement

IV. Déroulement de la RCP

Information du patient

Le patient doit être informé au préalable par son médecin référent que son dossier va être soumis à la RCP. Il s'engage à recueillir le consentement de son patient concernant l'échange et le partage de ses données. La présentation du dossier en RCP sera tracée dans le dossier du patient.

Modalités d'inscription

Tout médecin prescripteur doit saisir une fiche de passage en RCP sur l'outil RCP ROFIM au minimum deux jours avant la RCP.

La fiche RCP comprend les informations suivantes :

- Le nom du médecin référent et du médecin présentant le dossier (si différent)
- Les antécédents personnels et familiaux du patient
- Le résumé clinique du patient
- L'état fonctionnel actuel du patient
- L'évolution récente du patient
- Les traitements spécifiques suivis par le patient

Tous les documents et examens complémentaires jugés pertinents pourront être joints à la fiche RCP.

Toute fiche incomplète ou saisie hors délai pourra entrainer un refus de présentation à la séance prévue. Le caractère urgent d'un dossier pourra être invoqué et s'il est jugé valable, pourra permettre la discussion du dossier de façon prioritaire.

La liste des patients dont le dossier doit être discuté en RCP est disponible sur l'outil ROFIM. Dans le cas où tous les dossiers prévus à la séance, ne peuvent pas être discutés si la durée de la séance de la RCP ne le permet pas, les dossiers restants seront discutés de façon prioritaire à la RCP suivante. En l'absence de dossier prévu à une séance, celle-ci sera annulée la veille.

Déroulement de la séance

Le médecin coordonnateur de la RCP veille au respect des horaires, organise la prise de parole (ordre de présentation des dossiers, équité du temps de parole, pertinence, etc…). Les médecins présents à la RCP doivent respecter le rôle du modérateur de séance. Le médecin responsable de la séance doit s'assurer que le quorum est atteint et maintenu jusqu'à la fin de la séance. Les dossiers sont discutés successivement et de façon collégiale.

La discussion des dossiers s'appuie sur la fiche RCP, ainsi que tous autres documents nécessaires à la discussion des données (imagerie, photos, CR d'examens, etc…)

Liste de présence

Un relevé des présents est effectué par l'outil ROFIM. La liste des participants est conservée pour traçabilité.

Avis émis par la RCP

L’avis émis par la RCP doit être argumenté et remis dans son contexte. Pour cela, les informations suivantes devront être notées sur la fiche d’avis de RCP :

- Si la RCP s’appuie sur un référentiel ou des recommandations de Bonnes Pratiques Cliniques
- Si l’avis est réservé en raison de la qualité des données disponibles. En cas d’informations manquantes, le dossier devra être réexaminé.
- En cas de divergence, il est demandé au responsable de la RCP de Proposer deux avis principaux en les argumentant

La fiche RCP contenant l’avis sera téléchargeable sur l’outil ROFIM par les médecins ayant participé à la séance.

Le médecin ayant présenté le cas de son patient doit inscrire l’avis de la RCP dans le dossier du patient et si la décision de prise en charge diffère de celle proposée, alors, le médecin doit noter dans le dossier du patient la raison de son choix.

V. Bilan annuel d’activités de la RCP

Le médecin coordonnateur de la RCP organise une information annuelle consacrée au fonctionnement de la RCP, avec :

- Présentation des chiffres d’activités, en termes de nombre de séances, de nombre de participants, de nombre de dossiers soumis, de nombre de dossiers discutés
- Présentation des indicateurs qualité fixés dans la charte : délai de traitement d’une demande d’avis, respect du quorum, retour à 1 an de la décision prise par la RCP : patient traité non ou non, prise en charge en accord avec la RCP, …
- Synthèse sur les difficultés rencontrées
- Proposition d’actions d’améliorations

VI. Coordonnées

Médecins coordonnateurs de la RCP

Nom : Dr Emmanuelle Campana-Salort
Etablissement : Hôpital de la Timone, Marseille
Mail : emmanuelle.salort-campana@ap-hm.fr

Nom : Dr Pascal Cintas
Etablissement : Hôpital Pierre Paul Riquet, Toulouse
Mail : cintas.p@chu-toulouse.fr

ARC organisateur des RCP

Nom : Mélanie Brison
Etablissement : Hôpital Nord, Saint-Etienne
Mail : melanie.brison@chu-st-etienne.fr

ANNEXE : ENGAGEMENT DE CONFIDENTIALITÉ DES RÉUNIONS DE CONCERTATION PLURIDISCIPLINAIRE (RCP) – FILIÈRE DE SANTÉ MALADIES RARES FILNEMUS

Toute personne participant aux RCP FILNEMUS, quel que soit son statut, est tenue au secret professionnel. Afin de protéger la vie privée des patients et la confidentialité des données personnelles de santé, aucune information, directement ou indirectement nominative, discutée lors des RCP ne doit être communiquée à des tiers sans autorisation. Toute violation du présent engagement m'expose à des sanctions disciplinaires et pénales conformément à la réglementation en vigueur.

Je m'engage à :

- ne pas divulguer d'informations auxquelles j'ai eu accès durant ma participation à la RCP, en particulier les informations confidentielles concernant les personnes dont les cas sont discutés en RCP, à moins que ces personnes soient dûment autorisées, en raison de leurs fonctions, à en recevoir communication ;

- m'assurer, dans la limite de mes attributions, que seuls des moyens de communication sécurisés seront utilisés pour transférer ces informations.

Cet engagement de confidentialité, en vigueur pendant toute la durée de mes fonctions, demeurera effectif, sans limitation de durée après la cessation de mes fonctions, quelle qu'en soit la cause.

Je déclare avoir pris connaissance de la charte de fonctionnement ainsi que de l'engagement de confidentialité des réunions de concertation pluridisciplinaire FILNEMUS et m'engage à tout mettre en œuvre pour assurer la protection des données personnelles des patients dont les dossiers seront discutés en RCP FILNEMUS.

Fait à

le

Nom Prénom :

Signature précédée de la mention « lu et approuvé » :
