# Supplementary material for: Multidisciplinary team meetings in treatment of spinal muscular atrophy adult patients: a real-life observatory for innovative treatments
Source: Orphanet J Rare Dis. 2024 Jan 24;19:24. doi: 10.1186/s13023-023-03008-6 (PMC10809505; doi:10.1186/s13023-023-03008-6)
Supplement: Supplementary file 2 — Additional file 2. Minimum set of clinical information form required to submit a case to the SMDT. [file 13023_2023_3008_MOESM2_ESM.pdf]

SMDTs - FILNEMUS

Physician participating in the SDMTs

File presented by :<br><br>Referring physician (if other than above) :

**FILE TYPE**<br><br>☐ **Therapeutic discussion**<br><br>☐ **Evolution update**<br><br>☐ **Other:**

**Patient**			
**Name**		**Birthdate**	
**First name**		**Sex**	**F**☐ **M**☐
**Weight :**	**Height :**		
**Family history**			
**Personal history**			

**Disease history**	**Age at onset :**<br><br>**motor development**<br><br>**milestone achievement:**

	☐ **Stitting** ☐ **Walking with help**<br>☐ **Walking independently** ☐ **Running**<br><br>Genetic testing<br>SMN1 : deletions/ mutations ?<br>SMN2 : number of copy if available
	**Motor and respiratory assessment**
	☐ **Walk with help** ☐ **Noninvasive ventilation**<br>Age : Age :<br>☐ **Loss of walking** ☐ **Invasive ventilation**<br>Age : Précisez âge :<br>☐ **Spine fusion**
	**Clinical and paraclinical summary (10 lines maximum) if other relevant elements**

Current status and recent developments

**Lower limb involvement**<br>**Walking**<br>☐ **Normal**<br>☐ **Abnormal without cane**<br>☐ **With 1 cane**<br>☐ **With 2 canes or walker**<br>☐ **Wheelchair**<br>☐ **Electric wheelchair**<br>☐ **Grabataire**	Upper limb involvement<br>If yes :<br>☐ autonomy for feeding<br>☐ autonomy for toileting/dressing<br>☐ autonomy for driving<br>☐ autonomy for computer access<br>☐ Axial impairment:<br><br>☐ other information on the level of autonomy
**Respiratory involvement**<br>☐ **restrictive syndrome**<br>**Specify FVC in % :**<br>☐ **nocturnal NIV**<br>☐ **NIV nocturnal and diurnal**	☐ **professional activity**

☐ Specify number of hours / d :

☐ Invasive ventilation

Specify number of hours / d :

☐ other

Progression over a follow-up period: Specify follow-up period:

☐ Stable

☐ Worsening

Specify:

☐ Improvement

Specify:

In the table below, fill in the relevant follow-up information for the patient:

Date (month/ year)							
6MWT							
10m WT							
TUG							
MFM							
RULM							
Myotools							
FVC							
Other (specify)							

Specific treatment ongoing

☐ Nusinersen

Start date :

End date :

Tolerance problems :

Please specify:

☐ **Risdiplam:**

Start date :

End date :

Tolerance problems :

Please specify:

Other events<br><br>Specify: Other events<br><br><br>Evolution under treatment :	Other events<br><br>Specify: Other events<br><br><br>Evolution under treatment :
☐ **Salbutamol**<br>Start date :<br><br>End date :<br><br>Tolerance problems :<br><br>Please specify:<br><br>Other events<br><br>Specify: Other events<br><br><br>Evolution under treatment :	☐ **Other treatment**<br>Start date :<br><br>End date :<br><br>Tolerance problems :<br><br>Please specify:<br><br>Other events<br><br>Specify: Other events<br><br><br>Evolution under treatment :

Questions asked at the RCP ?

☐ Request for starting treatment

If yes, please specify:

☐ Nusinersen

☐ Risdiplam

☐ other

☐ Request for change of treatment

☐ Other

Please specify:

Advice of the SMDT (will be left in free fields but will be used as a guide to always reproduce the same scheme in case of approval of treatment)

☐ The SMDT approves the indication for treatment with (Nusinersen or Risdiplam)..... for this patient with / without reserves (if reserves, specify) due to...

The patient will be reassessed at x months of treatment.

The evaluation scales selected are

☐ 6-minute walk test

☐ **10m walking test**

☐ **TUG**

☐ **MFM**

☐ **RULM**

☐ **Myotools**

☐ **others: specify**

Discontinuation criteria are:

OR

☐ The SDMT does not approve to the treatment due to :

It is suggested:

**SMDT coordinator**		**Date**	
